# Acute kidney injury after intracerebral hemorrhage: a mini review

**DOI:** 10.3389/fmed.2024.1422081

**Published:** 2024-06-26

**Authors:** Yuyang Chen, Guang Zhao, Xiaohua Xia

**Affiliations:** Department of Emergency Medicine, Affiliated Kunshan Hospital of Jiangsu University, Kunshan, China

**Keywords:** intracerebral hemorrhage, acute kidney injury, pathogenesis, risk factors, diagnostic approaches

## Abstract

Intracerebral hemorrhage (ICH) stands as a prevalent and pivotal clinical condition. The potential cooccurrence of acute kidney injury (AKI) among afflicted individuals can profoundly influence their prognosis. In recent times, there has been a growing focus among clinical practitioners on researching the relationship between ICH and AKI. AKI occurring concurrently with ICH predominantly arises from both hemodynamic and non-hemodynamic mechanisms. The latter encompasses neurohumoral regulation, inflammatory response, oxidative stress, and iatrogenic factors such as contrast agents, dehydrating agents, antibiotics, and diuretics. Moreover, advanced age, hypertension, elevated baseline creatinine levels, chronic kidney disease, and larger hematomas predispose patients to AKI. Additionally, the current utilization of biomarkers and the development of predictive models appear promising in identifying patients at risk of AKI after ICH. This article aims to underscore the potential of the aforementioned insights to inspire novel approaches to early clinical intervention.

## Background

Intracerebral hemorrhage (ICH) represents a subtype of stroke, comprising 15–30% of all stroke cases (1, 2). The prognosis for individuals affected by ICH is frequently bleak, with many experiencing residual neuropsychiatric symptoms that hinder daily functioning. Studies have documented the mortality rate of ICH as elevated as 50% (3). Acute kidney injury (AKI) is a frequent clinical complication in individuals with ICH. However, due to variations in AKI definitions and differences in study populations, the reported incidence of AKI in ICH patients varies considerably. Studies indicated an incidence ranging from approximately 15–40% (4–6), with the incidence of AKI necessitating dialysis reaching up to 3.5% (7). Patients experiencing AKI following ICH demonstrate elevated mortality rates and a heightened incidence of neurological deterioration compared to those without AKI (8, 9). Therefore, comprehending the pathophysiological mechanisms, risk factors, and current methodologies for AKI diagnosis in individuals with ICH may hold significant importance for patient prognosis.

## The pathological mechanisms of AKI in ICH patients

**Figure 1:**
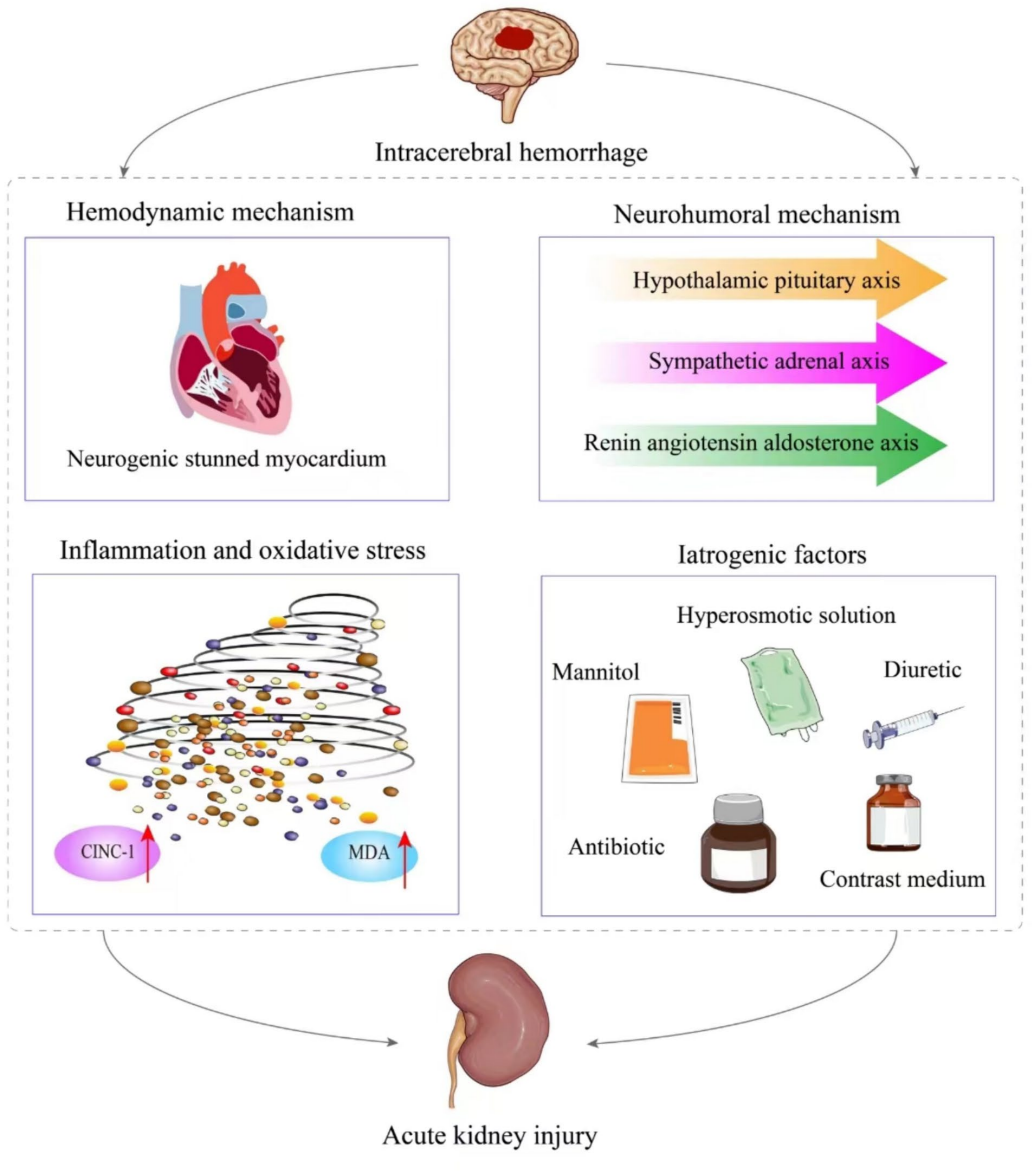
The pathological mechanism of acute kidney injury after intracerebral hemorrhage. It is mainly divided into hemodynamic mechanism and non-hemodynamic mechanism. The latter includes neurohumoral mechanism, inflammation and oxidative stress, as well as iatrogenic factors.

The pathophysiological mechanisms of AKI following ICH are complex, involving both hemodynamic and non-hemodynamic factors that interact and promote each other. Non-hemodynamic mechanisms include neurohumoral mechanisms, inflammation and oxidative stress, and iatrogenic factors such as the use of contrast medium, mannitol, and nephrotoxic drugs. We primarily discuss widely accepted pathophysiological mechanisms (Figure 1). However, the exact mechanisms remain inconclusive, and further experimental research is needed to explore these potential pathways.

## Hemodynamic mechanisms

ICH can induce neurogenic stunned myocardium. Intracranial pressure increases during brain injury, affecting the right insular cortex/Amygdala Hypothalamus, leading to autonomic nervous dysfunction and significant sympathetic system activation. Cardiomyocytes release excessive catecholamines, which open calcium channels and cause contraction band necrosis and myocyte dysfunction (10). This results in neurogenic stunning of the myocardium, leading to kidney injury through two main pathways. Firstly, myocardial cell damage and heart pump function failure lead to a reduction in cardiac output and impaired blood circulation. This compromised circulation fails to meet the normal renal perfusion requirements, resulting in kidney ischemia, hypoxia, and subsequent kidney injury. Moreover, to prioritize blood perfusion to vital organs like the brain and lungs, the renin-angiotensin-aldosterone system is activated. This activation leads to renal artery constriction, exacerbating the decrease in renal perfusion and contributing to the development of AKI (11). Secondly, neurogenic stunned myocardium after intracerebral hemorrhage can lead to congestive heart failure, with or without pulmonary edema (12). Blood stasis in the pulmonary capillary network increases vena cava pressure, blocks venous return, causes renal vein congestion, and decreases the glomerular filtration rate (13).

## Non hemodynamic mechanisms

### Neurohumoral mechanisms

When patients experience ICH, activation of the hypothalamic–pituitary axis, sympathetic-adrenal axis, and renin-angiotensin-aldosterone axis occur. Animal models have further substantiated this by demonstrating a significant increase in cortisol, adrenaline, and angiotensin II levels in collagenase-induced ICH mice, indicating activation of these three systems during ICH (14, 15). Additionally, vasoconstrictors are present, contributing to the constriction of glomerular arterioles and enhancing renal tubular reabsorption, ultimately leading to a decrease in glomerular filtration rate (GFR) (16).

### Inflammatory and oxidative stress

In an experiment inducing ICH in rats with collagenase, notable increases were observed in the levels of cytokine-induced neutrophil chemoattractant-1 (CINC-1) and malondialdehyde (MDA) in urine. They also detected the presence of podocalyxin DNA, indicative of podocyte detachment (17). CINC-1 serves as a neutrophil chemotactic agent, recruiting concentrated granulocytes to trigger an inflammatory cascade response in distant organs such as the kidney (18). MDA represents the primary stable product following oxidative stress and has been extensively implicated in the pathogenesis of kidney injury (19). Hence, these findings suggest the involvement of inflammation and oxidative stress in disrupting podocyte function, consequently contributing to AKI. Additionally, the authors postulated that activation of the NF-κB pathway may also contribute to kidney injury (17). However, further fundamental research is warranted to elucidate the specific mechanism.

### Iatrogenic factors

#### Contrast medium

Etiological identification in ICH patients often necessitates computed tomography angiography (CTA) examination for diagnostic assistance. However, the use of contrast medium poses a significant concern for AKI in such patients. The renal damage caused by contrast medium has been internationally recognized (20), particularly concerning high-osmolarity contrast medium. Close monitoring of renal function and urine output is imperative. The American Radiological Society recommended administering physiological saline hydration therapy before and after contrast medium administration to mitigate the risk of AKI (21). Nonetheless, a study reported that the incidence of AKI in ICH patients after CTA was merely 25%, and correlation analysis indicated no association between AKI and CTA performance (*p* = 0.187) (22). Another study also found no significant relationship between contrast medium use in CTA and AKI [Odds Ratio (OR) = 1.16, 95%Confidence interval (CI): 0.72–1.95, *p* = 0.5548] (23). This may be attributed to the widespread use of low-or iso-osmolar contrast medium in clinical practice, as well as the relatively small doses administered during CTA. Nevertheless, clinical vigilance regarding the frequency and dosage of contrast medium administration remains paramount, especially for patients with preexisting chronic kidney disease (CKD).

#### Mannitol and hypertonic solution

Patients with ICH frequently present with elevated intracranial pressure. Currently, common drugs used in clinical practice include mannitol and hypertonic sodium solutions. A multicenter study involving 9,649 cases of ICH demonstrated that mannitol significantly contributes to AKI (OR = 1.986, 95%CI: 1.426–2.813, *p* < 0.001) (24). Mannitol initially induces renal vasoconstriction, leading to inadequate renal perfusion. Subsequently, its robust diuretic effect may precipitate volume depletion, alter blood flow distribution, and culminate in renal medullary ischemia and hypoxia (25). Moreover, it can cause swelling of renal tubular cells, resulting in osmotic nephropathy (26). Additionally, hypernatremia and hyperchloremia induced by hypertonic solutions may also correlate with AKI (27). A study on the rapid infusion of a high-sodium solution in dogs demonstrated that despite the decrease in renin secretion, concurrent contraction of renal artery and a reduction in GFR were observed. Authors proposed that this vasoconstriction might be independent of renin and constitutes a “tubular feedback” mechanism of kidneys toward blood sodium (28). Consequently, hypernatremia from hypertonic solutions could induce kidney damage (29). Hyperchloremia is also a significant cause of AKI in ICH patients. Yunos et al. demonstrated that restricting treatment with chlorine-containing drugs significantly reduced creatinine levels compared to the control group (14.8 μmol/L vs. 22.6 μmol/L, *p* = 0.003). Notably, it also decreased the incidence of AKI (8.4% vs. 14%, *p* < 0.001) and dialysis rates (10% vs. 6.3%, *p* = 0.005) (30). Studies have revealed that a variety of vasoactive substances can activate chloride ion channels in vascular smooth muscle cells through calcium ions, inducing chloride ion efflux, cell membrane depolarization, and subsequent calcium ion influx, ultimately leading to vasoconstriction (31). Hence, chloride ions can induce constriction of the afferent arterioles, resulting in diminished renal perfusion and a decline in GFR. Additionally, chloride ions can elevate thromboxane synthesis, further constricting renal blood vessels (32). Furthermore, studies have indicated that hyperchloremia is a significant factor contributing to the delayed resolution of cerebral edema (OR = 5.24, 95%CI: 1.64–16.76) (33). Accordingly, certain scholars have suggested that in cases of ICH, proactive management of blood sodium and chloride levels may help mitigate the incidence of AKI and mortality (5, 34).

#### Nephrotoxic drugs

Some nephrotoxic drugs commonly used in clinical practice can also induce AKI in patients with ICH. Among these, antibiotics such as vancomycin and diuretics are frequently implicated. She et al. utilized the random forest algorithm and found that vancomycin emerged as a significant predictive factor for AKI in patients with ICH (35). Additionally, another study noted that diuretic escalated the risk of AKI in individuals with acute stroke (OR = 8.5, 95%CI: 3.46–20.86, *p* < 0.001) (26). This phenomenon could be attributed to the capacity of diuretics to alter renal microcirculation, potentially leading to renal ischemia, hypoxia, and inflammatory reactions, ultimately resulting in AKI. Consequently, it is imperative to judiciously select nephrotoxic drugs in clinical settings to mitigate adverse renal outcomes in ICH patients. Figure 1 shows the relevant mechanisms of acute kidney injury after intracerebral hemorrhage.

## Risk factors for AKI in ICH patients

In patients with ICH, the risk of AKI varies. It is essential to understand the clinical characteristics of patients susceptible to AKI, including specific risk factors. This understanding can assist clinical practitioners in identifying high-risk populations for AKI among patients with ICH.

### Age

Advanced age is often regarded as one of the risk factors for AKI in patients with ICH. Zhang et al.’s study on ICH patients revealed that age serves as an independent predictor of AKI, irrespective of whether the patient has CKD or not (*p* = 0.002 and *p* = 0.008) (36). Another retrospective study similarly discovered that ICH patients aged over 70 years are at higher risk of AKI (OR = 14.652, 95%CI: 1.397–153.634, *p* = 0.003) (37). This susceptibility in older patients may stem from insufficient residual renal units, rendering them more vulnerable to AKI triggers such as ischemia, hypoxia, inflammatory stimuli, and oxidative stress. Given the advanced age of patients with ICH, some researchers have posited that the association between age and AKI remains uncertain (38). Hence, large-scale clinical studies and fundamental experiments are warranted to provide further confirmation.

### Hypertension

Hypertension, as a significant risk factor for AKI in patients with ICH, has been extensively documented. A retrospective study found that the incidence of AKI increased with the level of blood pressure at admission (mild, moderate, and severe: 28.2, 37.1, 56%, *p* < 0.001), with severe hypertension closely associated with AKI (OR = 2.6, 95%CI: 1.5–4.3, *p* < 0.001) (39). Furthermore, the magnitude of blood pressure reduction is also linked to AKI. Tanaka’s study demonstrated that the risk of AKI was significantly higher in the group with a high to low systolic blood pressure reduction compared to the group with a medium to low reduction (OR = 3.50, 95%CI: 1.83–6.69, *p* < 0.01) (40). Another study also observed a correlation between systolic blood pressure reduction and AKI (9). On one hand, hypertension can lead to kidney damage due to endothelial dysfunction in arteries (41). On the other hand, hypertensive patients have a high demand for renal perfusion pressure, which can result in renal ischemia and hypoxia when there is a rapid and significant reduction in blood pressure (42).

### Elevated baseline creatinine and CKD

Qureshi’s research indicated that patients with ICH who had a baseline blood creatinine level ≥ 110 μmol/L faced an elevated risk of AKI and renal adverse events, with OR of 2.4 (95%CI, 1.2–4.5) and 3.1 (95%CI, 1.2–8.1), respectively (4). A study involving 1,366 ICH patients similarly highlighted creatinine as a characteristic of AKI (35). However, some studies have found no correlation between renal function and AKI, and the presence of CKD alongside AKI did not correlate with long-term mortality (OR = 3.13, 95%CI: 0.65–15.01, *p* = 0.154) (43). The author posited that this may be due to the resistance of patients with CKD to kidney damage. However, in reality, the mortality rate in the later stages of CKD significantly increased (41). This underscores the importance for clinicians to remain vigilant in monitoring the renal function of ICH patients. Indeed, certain risk factors for AKI in patients with ICH are commonly observed in ischemic stroke as well, including significant blood pressure fluctuations and chronic kidney disease (44, 45). This indicates that the treatment of ICH should avoid aggravating kidney injury by causing cerebral ischemia.

### Large intracranial hematoma

Burgess et al.’s study revealed that compared to non-AKI patients, those with AKI presented with a larger hematoma volume upon admission (15.5 ± 17.8 vs. 12.0 ± 14.0, *p* = 0.045). Patients with larger hematoma volumes may experience more pronounced activation of the sympathetic nervous system, potentially leading to kidney damage through the neurohumoral regulatory mechanisms mentioned earlier (43). Furthermore, there are reports indicating abnormal coagulation function in ICH patients, which could contribute to hematoma enlargement. Clinicians should therefore closely monitor the patient’s coagulation status and judiciously select medications to mitigate the risk of kidney injury (46).

## Early prediction of AKI in ICH patients

### Biomarkers

#### Cystatin C

Cystatin C (CysC) in renal injury has been widely acknowledged (47). Jiang’s study demonstrated that the level of CysC could more accurately predict the occurrence of AKI in patients with acute stroke [Area under the Receiver Operating Characteristic Curve (AUC) = 0.772, 95%CI: 0.726–0.813], surpassing traditional creatinine (AUC = 0.660, 95%CI: 0.610–0.708) (38). Additionally, CysC level were associated with the mortality prognosis of ICH patients (48). Hence, CysC may serve as a more comprehensive biomarker.

#### β2-microglobulin

β2-microglobulin (β2-MG) is a peptide unaffected by gender or age that serves as a reflection of renal function. A prospective study involving 403 ICH patients revealed that β2-MG exhibited strong predictive efficacy for AKI, with an AUC of 0.712 (95%CI: 0.652–0.772). Additionally, ICH patients with β2-MG levels exceeding 2123.50 mg/L faced a significantly elevated risk of developing AKI (OR = 2.606; 95%CI: 1.315–5.166), which was also associated with in-hospital and 1-year mortality (49). These findings suggest the potential for β2-MG to emerge as a novel predictive indicator.

#### Procalcitonin

Procalcitonin (PCT) is a widely used indicator in clinical practice to assess infection and inflammation. However, a recent study suggested that PCT could also serve as a risk factor for AKI in non-sepsis patients (OR = 4.430, 95%CI: 1.464–13.399) (50). In studies related to ICH, a PCT value exceeding 0.5 μg/L upon admission was identified as a significant predictor for dialysis in ICH patients (OR = 7.7, 95%CI: 1.4–43.3, *p* = 0.02) (51), and is independently associated with poor prognosis and 3-month mortality (52). This could be attributed to the activation of immune cells by pro-inflammatory products generated during ICH and kidney injury, leading to an exacerbation of the inflammatory response and subsequent elevation of PCT levels in the patient’s blood (53). Given the widespread use of PCT in clinical practice, understanding its predictive role for AKI in ICH patients is crucial for optimizing medical resource allocation.

## Prediction model

Presently, research on predictive models for AKI in ICH patients is highly significant. Tian et al. utilized the least absolute shrinkage and selection operator (LASSO) in conjunction with multivariate logistic regression methods to construct a model comprising 9 clinical and laboratory examination features. Through internal and three external validations, they demonstrated the model’s strong predictive performance, with AUCs of 0.816, 0.776, 0.780, and 0.821, respectively (24). Furthermore, Lin et al.’s acute stroke model exhibited notable predictive efficacy, reaching up to 0.839 (26). In addition, She et al. used six machine learning algorithms—extreme gradient boosting, logistic, light gradient boosting machine, random forest, adaptive boosting, and support vector machine—to develop the model. They found that the random forest algorithm performed the best in both the training and validation sets, achieving an AUC of 1.000 in the training set and 0.698 in the validation set (35). However, these models are constrained by relatively small sample sizes, limiting their credibility. Understanding the timing of AKI occurrence in patients is crucial for facilitating early clinical intervention. Furthermore, the practicality of the model for clinical application should be considered, including the development of simple scoring tables or prediction web platforms. Therefore, further research is imperative to refine and enhance these models.

## Conclusion

Patients with ICH are critically ill, and AKI represents a significant clinical complication for them. Those who develop AKI often face a poor long-term prognosis. Currently, there is no specific treatment for AKI following ICH. Therefore, understanding the mechanisms, risk factors, and early diagnosis of this type of AKI is crucial. Further basic and clinical research is needed to improve our understanding of the disease. In the future, we aim to develop better models to identify high-risk patients and create new interventions to improve patient prognosis.

## Author contributions

YC: Conceptualization, Formal analysis, Investigation, Methodology, Supervision, Validation, Writing – original draft, Writing – review & editing. GZ: Conceptualization, Funding acquisition, Methodology, Writing – review & editing. XX: Conceptualization, Formal analysis, Funding acquisition, Methodology, Supervision, Writing – review & editing.
